# An overview of PLOS curated collection in health literacy

**DOI:** 10.1371/journal.pone.0339159

**Published:** 2026-01-06

**Authors:** Rubeena Zakar, Sarosh Iqbal

**Affiliations:** 1 Department of Public Health, University of the Punjab, Lahore, Punjab, Pakistan; 2 Department of Sociology, Forman Christian College University, Lahore, Punjab, Pakistan; Public Library of Science, UNITED KINGDOM OF GREAT BRITAIN AND NORTHERN IRELAND

## Abstract

This curated collection, launched in 2022 and concluded in 2024, explores the critical role of health literacy (HL) as a determinant of health, particularly within the framework of the 2030 Agenda for Sustainable Development Goals (SDGs). Despite not being explicitly included in the SDGs, HL underpins efforts to achieve Universal Health Coverage by empowering individuals to make informed healthcare decisions. The collection encompasses 22 original articles employing diverse methodological approaches across global settings, highlighting themes such as HL’s influence on health outcomes, access to healthcare, effective interventions, and policy integration. The articles illustrate that adequate HL is essential for navigating complex healthcare landscapes, particularly for vulnerable populations disproportionately affected by low HL. Challenges associated with inadequate HL contribute to poor self-care behaviors, increased health risks, and diminished healthcare utilization. The collection emphasizes the importance of measuring HL through established tools and discusses the intersection of HL with social determinants of health. Furthermore, it addresses the need for targeted HL interventions, particularly in marginalized communities, and underscores the role of policymakers in embedding HL into public health strategies. Ultimately, this collection provides a comprehensive overview of HL’s multifaceted nature, its implications for individual and public health, and the urgent need for sustained investment in HL research and interventions. By fostering improved HL, we can enhance healthcare access, improve health outcomes, and reduce health disparities globally.

## Introduction

The 2030 Agenda for Sustainable Development, particularly Sustainable Development Goal-3 (SDG 3), focuses on ensuring healthy lives and promoting well-being for people of all ages. This goal is closely linked with the broader vision of achieving Universal Health Coverage (UHC). Attaining this goal requires that individuals have the ability to access, understand and use health information effectively – a capability defined as health literacy (HL) [1]. HL is crucial because it empowers individuals to make informed decision-making about their healthcare by enabling them to obtain, comprehend, evaluate, and apply health-related information and services [1]. Although HL is not explicitly included in the SDGs, its principles are essential to empowering individuals to obtain quality healthcare, advancing the broader goals of SDG-3 and UHC, and promoting health equality and population health [2].

The term ‘health literacy’ was coined in the 1970s [3] to emphasize the importance of understanding health education and its impact on individuals’ abilities to make informed health decisions. HL highlights the interconnected roles of healthcare and educational institutions in promoting better health outcomes [3]. Various models of HL, including functional, interactive, and critical HL have been introduced to examine the relationship between HL and these institutions [4,5]. Particularly in the early 2000s, HL research expanded to explore its effects on socio-cultural dynamics within healthcare and educational institutions [6]. During COVID-19 and post-pandemic period, HL has gained renewed attention as a crucial factor across all age groups, highlighting the need for improved HL to enhance quality of life [7,8]. As a result, HL has evolved into a multidimensional concept and a key indicator for a well-functioning healthcare system, influencing health education and policy reforms [8].

Adequate HL is pre-requisite for individuals in navigating today’s complex healthcare landscape to effectively address their health needs. HL equips individuals with the knowledge, competencies, and skills necessary for disease prevention and health promotion, empowering them to lead healthier and higher quality lives [8]. Conversely, individuals with inadequate or limited HL often face significant challenges, such as poorer self-care, reduced use of preventive measures, adverse health outcomes, and an increased risk of mortality [9]. Particularly, vulnerable and marginalized groups, such as women, refugees, older adults, and individuals with lower socioeconomic status, are affected disproportionately. Additionally, inadequate HL negatively influences healthcare utilization, patient-provider relationships, and overall patient satisfaction [10].

Recognizing the significance of HL, the Public Library of Science (PLOS) launched curated collection on HL (https://collections.plos.org/collection/health-literacy/) [11] in 2024 following selection of articles published across various PLOS journals over the previous two years (2022–2024). This collection comprises of 22 original articles, contributed by global researchers. These studies offer a diverse range of methodological approaches and examine HL across various geographical regions worldwide. Methodologically, this collection presents multiple research designs, encompasses scoping and systematic reviews, mixed-method research, randomized controlled trials, quantitative surveys, and qualitative interviews and discussions. Geographically, they cover Europe, North America, Africa, Asia, and Oceania, illustrating the universal relevance of HL. Despite the diversity in settings, these studies consistently demonstrated a positive association between higher HL and improved health outcomes.

On a whole, this collection can be categorized into the following four cross-cutting themes: 1) HL as a determinant of health; b) HL and access to healthcare; c) HL interventions; and d) HL and policy integration. Before exploring these themes, it is essential to highlight the measurement of HL, which emerged as a pertinent area of focus within this collection.

## Measurement of HL

Measurement of HL is crucial, as adequate HL serves as a protective factor for a higher quality of life. Several tools have been developed over time to measure HL, including the European Health Literacy Survey Questionnaire, which is used in this collection [12,13]. These tools assessed individuals’ HL and its association with their quality of life. While these tools provide valuable insights into HL, it is important to recognize their limitations and the need for cultural sensitivity in their application. Moreover, adapting these tools to diverse populations and healthcare setting is imperative to ensure their accuracy and relevance in measuring HL on a global scale. This consideration enhances the validity of HL assessments and support more effective cross cultural comparisons.

### 1. Health literacy as a determinant of health

HL is closely intertwined with social determinants of health, such as age, education, employment, and socioeconomic status [14–19]. For example, Riiser et al. explored HL among adolescents and its impact on their quality of life [14]. Chidambaram et al. further highlighted the role of socio-demographic, economic, and political factors in shaping individuals’ interactions with the digital healthcare system and building digital HL, also known as digital determinants of health (DDOH) [16]. Another study by Arias López and colleagues emphasized the importance of digital HL in the era of modern technology for better self-management, and participation in healthcare decision-making to improve quality of life [15].

HL-related knowledge and skills enable individuals to identify health risks, adopt protective measures, and effectively manage their health problems. Improved HL empowers individuals to make informed choices that support their well-being. For example, Miyoshi & Watanabe’s path analysis explored how genomic knowledge, health numeracy, and interactive HL predict individuals’ risk perception and healthcare decisions for treatment [20]. Additionally, Sargsyan et al. accentuated the effects of socio-demographic factors on HL, found that these factor shape health knowledge and behavior in adopting better preventive practices [19]. Furthermore, Bailey et al. highlighted that a lack of knowledge about medication overdoses, especially among older adults and minorities, indicates low HL, leading to adverse health outcomes and medical complications [21]. These findings underscore how HL shapes personal health choices, health behavior, and their consequences.

Individuals from underdeveloped backgrounds often have limited opportunities for education, employment, and financial stability, contributing to lower HL. Conversely, individuals with higher HL tend to engage in healthier behaviors, such as preventive screenings, a healthy diet, and regular exercise [17,18]. Higher HL is imperative for older age groups and individuals with chronic diseases like diabetes and hypertension to reduce mortality and promote healthy outcomes [17,18]. Moreover, studies from Sweden [22] and Switzerland [23] highlight that low HL disproportionately affects migrant refugees and socioeconomically disadvantaged individuals [22,23]. Due to social exclusion and marginalization, these groups face additional barriers to accessing healthcare information and services, including language barriers, limited education and employment opportunities, fewer housing facilities, and cultural differences. Financial deprivation is a critical factor contributing to marginal HL, leading to poorer health experiences, such as increased hospitalization rates and decreased adherence to treatment regimens.

### 2. Health literacy and access to healthcare

Limited HL significantly hinders individuals’ access to necessary healthcare services. This can lead to difficulties in navigating the complex hierarchy of healthcare systems, understanding medical terminologies, and prescriptions, and managing health conditions [24,25]. These challenges may result in delayed diagnosis, inappropriate or untimely treatment, and deteriorated health outcomes, consequently exacerbating health inequalities. This is particularly evident in the case of differently-abled individuals, e.g., the deaf community, who face significant barriers in accessing healthcare information and services, often leading to suboptimal health outcomes [26].

Disparities in education, healthcare access, and digital resources also affect HL levels. Epidemiological and cluster analyses revealed that younger, educated individuals tend to have higher HL compared to older, less-educated individuals, creating structural barriers. Nam & Yoon emphasized the mediating role of access to healthcare, provider-patient interaction, motivation, self-efficacy and self-care behavior among diabetes patients with physical disabilities, highlighting how HL can improve patient autonomy and health management [25].

Additionally, parents’ limited knowledge coupled with digital HL emerge as a significant barrier to access to healthcare services. For instance, parents’ ability to assess vaccine-related health information can significantly impact their decision-making regarding vaccine uptake for their children [27]. However, trust in healthcare providers remains a crucial factor for parents’ healthcare decisions.

Another dimension of healthcare access relates to healthcare professionals, who play a pivotal role in improving HL through health education and shared decision-making opportunities [28]. Healthcare providers can enhance HL and health education by using simple language, avoiding medical jargon, and actively listening to their patients’ concerns to facilitate informed decision-making. Improved self-perceived health is closely linked to higher HL. To address these challenges, targeted interventions aimed at improving access to healthcare for vulnerable populations are essential.

### 3. Health literacy-related interventions

Promoting HL is crucial for individuals’ informed participation in healthcare and reducing health inequalities. However, various challenges hinder HL, necessitating interventions to address these challenges and propose effective solutions. Several interventions have been developed to improve HL, including educational programs, digital tools, and community-based initiatives [29–31]. Digital learning resources provide accessible and engaging ways for individuals to acquire health information and develop critical thinking skills. Hall et al. highlighted the role of visual and pictorial communication in enhancing HL and influencing individuals’ behavior change [30]. Furthermore, a culturally sensitive approach from healthcare providers can facilitate effective communication and understanding among patients from diverse backgrounds.

To improve HL of marginalized communities, Hosking et al. and Windfall et al suggested that school-based peer education and training of local community health workers can be highly effective [31,32]. Community health workers, being indigenous and culturally sensitive, can provide culturally relevant health information and support, effectively bridging the gap between healthcare providers and patients. It’s pertinent to mention that most studies in this curated collection recommend targeted interventions to improve the HL of the general population and patients to maintain a better quality of life.

In the modern era, the increased use of digital technology in healthcare has led to the emergence of digital HL, which refers to the ability to access, understand, and utilize digital health information and tools [16]. As our world becomes increasingly digitized, it is imperative for all individuals to develop the necessary knowledge, competencies, and skills to effectively navigate digital health resources.

### 4. Health literacy and policy integration

Local governments and healthcare systems play a crucial role in addressing HL challenges, with a focus on integrating HL into public health policies and programs [33]. Implementation of health promotion and HL initiatives within the educational system has the potential to build and enhance HL [33]. However, there is a pressing need to establish strong policy foundations for HL and embed its significance into the strategic documents and operational plans of healthcare systems.

Notably, this collection informed various challenges in implementing effective HL policies, including resource limitations, disparities in digital access, and socio-political factors [16]. In some settings, cultural barriers and language differences may hinder the efficacy of HL interventions [15,26,32].

The role of government and policymakers is pivotal in raising awareness about HL, securing adequate funding, promoting health equity, and improving positive health outcomes across all age groups. Initiatives such as school-based programs can help to improve HL among younger populations. There is also a need to develop a robust evaluation framework and mechanisms to measure the impact of HL interventions.

## Conclusion

HL is a global phenomenon and a critical determinant of health with significant implications for individual well-being and public health. In the context of increasingly digitalized healthcare systems, digital HL has become especially important. HL curated collection is a useful resource to build HL component of different dimensions of health. It can help researchers in operationalizing and linking HL with social determinants of health. Many studies in the collection are set in resource poor or low-middle income settings [24] which can guide researchers to use them as comparators when discussing their country context. Some papers in the collection developed or validated health literacy scales [12] which can be utilized by the future research on HL. The empirical studies from this collection can be used to strengthen arguments for investing in health literacy (especially in underserved, women, and minority populations).

Overall, this collection provides valuable insights into the complex relationship between HL, social determinants of health, barriers to access, and health outcomes. A multi-dimentional approach is required to address the pressing challenges related to HL, including investment in education, improved healthcare access, and the promotion of digital HL initiatives, particularly for marginalized populations. By addressing the challenges of marginal HL, we can improve healthcare access, enhance patients’ outcomes, and reduce health inequalities. Continued investment in HL research and intervention is essential to ensure that everyone has the opportunity to lead healthy lives and make informed decisions for quality life. Healthcare providers are seen as key players in promoting HL through effective communication and patient education. Additionally, the importance of cultural competence in HL interventions is emphasized. Strong policy frameworks and supportive systems are essential to address HL challenges and promote digital health initiatives. Through understanding these key lessons, we can work towards improving HL and empowering individuals to make informed decisions towards their health.
